# Sympathetic innervation in skeletal muscle and its role at the neuromuscular junction

**DOI:** 10.1007/s10974-024-09665-9

**Published:** 2024-02-17

**Authors:** Rüdiger Rudolf, Isis C. Kettelhut, Luiz Carlos C. Navegantes

**Affiliations:** 1 Center for Mass Spectrometry and Optical Spectroscopy, Mannheim University of Applied Sciences, 68163 Mannheim, Germany; 2 Interdisciplinary Center for Neurosciences, Heidelberg University, 69117 Heidelberg, Germany; 3 Mannheim Center for Translational Neuroscience, Medical Faculty Mannheim Heidelberg University, 69167 Mannheim, Germany; 4 Department of Biochemistry & Immunology, Ribeirão Preto Medical School, University of São Paulo, Ribeirão Preto-SP, 14049900 Brazil; 5 Department of Physiology, Ribeirão Preto Medical School, University of São Paulo, Ribeirão Preto-SP, 14049900 Brazil

**Keywords:** Acetylcholine receptor, Adrenoceptor, Neuromuscular junction, Sympathetic

## Abstract

Neuromuscular junctions are the synapses between motor neurons and skeletal muscle fibers, which mediate voluntary muscle movement. Since neuromuscular junctions are also tightly associated with the capping function of terminal Schwann cells, these synapses have been classically regarded as tripartite chemical synapses. Although evidences from sympathetic innervation of neuromuscular junctions was described approximately a century ago, the essential presence and functional relevance of sympathetic contribution to the maintenance and modulation of neuromuscular junctions was demonstrated only recently. These findings shed light on the pathophysiology of different clinical conditions and can optimize surgical and clinical treatment modalities for skeletal muscle disorders.

## Enigmatic links between sympathetic nervous system and skeletal muscle

Since the first days of modern research on skeletal muscle and neural physiology, the neuromuscular junction (NMJ) has been of prime interest and served as a model system for several fundamental principles of neuroscience, including the measurement of axonal conduction velocity (von Helmholtz 1852) and the establishment of the concepts of synapse (Sherrington 1906), quantal hypothesis (Katz 1971), synaptic vesicle recycling (Heuser and Reese 1973), and single channel recordings using patch-clamp technique (Neher et al. 1978). In general, the NMJ has often been considered as the classical example of a tripartite synapse, composed of a motor neuronal presynapse, a skeletal muscle postsynapse, and a capping by terminal Schwann cells (Sanes and Lichtman 2001; Engel and Franzini-Armstrong 2004). However, stimulatory and synchronizing effects of catecholamines, epinephrine and norepinephrine, on muscle contraction and quantal release (Kuba 1970; Khuzakhmetova and Bukharaeva 2021; Bukharaeva et al. 2021) suggested a function of sympathetic innervation in skeletal muscle and potentially at the NMJ. Further, several different autonomic disorders, such as congenital insensitivity to pain (Shorer et al. 2013), adrenal insufficiency (Martin-Grace et al. 2020), complex regional pain syndrome, and Lambert-Eaton myasthenic syndrome, share traits of muscle weakness. Finally, a correlation was observed between positive or negative pharmacological tuning of adrenoceptors and patients’ symptoms in NMJ-specific disorders, such as myasthenia gravis (Cao et al. 2020; Trillenberg et al. 2021) and some congenital myasthenic syndromes (Liewluck et al. 2011; Burke et al. 2013; Lorenzoni et al. 2013). Together, these data suggested an involvement of the sympathetic nervous system in the physiology of skeletal muscle and the NMJ.

## Classical functions of the sympathetic nervous system

The Sympathetic Nervous System (SNS) of vertebrates contributes to the maintenance of homeostasis through the release of catecholamines, which modulate a variety of physiological processes and behaviors. While epinephrine is secreted into the bloodstream by the adrenal glands and acts systemically as a hormone, norepinephrine is the principal neurotransmitter released by sympathetic neurons that innervate peripheral organs and tissues throughout the body. The contacts between sympathetic neurons and their targets are established during embryonic and postnatal development (Scott-Solomon et al. 2021) and are essential for controlling diverse physiological processes, including cardiac output, blood pressure, body temperature, glycemia and immune function under basal and stress conditions. Indeed, stressful stimuli activate the SNS which maintains the animal in an excited and alerted state (Goldstein 2010) and induces a metabolic and behavioral adaptation, leading to enhanced energy supply and increased muscle performance. This so-called “fight or flight” response is also observed in invertebrates. For instance, under food deprivation conditions, Drosophila larvae exhibit increased locomotor speed and synaptic bouton numbers at NMJs. Octopamine, the invertebrate counterpart of norepinephrine, plays critical roles in these processes, but the underlying mechanisms are still poorly understood (Kamimura et al. 2019). Numerous studies, reviewed in (Navegantes et al. 2002; Roatta and Farina 2010; Berdeaux and Stewart 2012) indicate that the SNS in mammals exerts anabolic actions on skeletal muscle that are important for the preservation of tissue structure and function. The majority of the adrenergic effects on skeletal muscle mass is mediated by β_2_-adrenoceptors (β_2_-ARs) which are the predominant subtype in skeletal muscle and localized with greater density on the sarcolemma of type-I rather than type-II muscle fibers (Lynch and Ryall 2008). β_2_-ARs can be pharmacologically targeted by specific agonists (e.g., clenbuterol and formoterol) which are the most heavily abused substances among bodybuilders and amateur fitness athletes seeking leanness (Jessen et al. 2020). In summary, the SNS adjusts skeletal muscle physiology under acute stress and it is critical for long-term maintenance of muscle.

## Morphological evidence supporting sympathetic innervation of NMJ

For many years, it was believed that the sympathetic innervation and adrenoceptors on skeletal muscle from mammals were restricted to vascular smooth muscle regulating muscle blood flow and blood pressure at rest and during exercise (DeLorey 2021). However, anatomical studies in the late 19th and the early 20th century using gold and silver staining of diverse muscles from different species suggested the presence of unmyelinated, thin nerve fibers and their extension into netlike structures in the immediate vicinity of NMJs (Bremer 1882; Boeke 1909, 1913; Agduhr 1920; Hines 1931). Based on their morphology, these fibers and endings were suggested to be of sympathetic origin, but clear experimental evidence for that was missing. After disputes, these findings were neglected for decades. Immunohistochemical and immunoelectron microscopic studies in the 1980ies detected tyrosine hydroxylase (TH), a marker for sympathetic neurons, in skeletal muscle fibers (Barker and Saito 1981) and more specifically, at the position of NMJs (Chan-Palay et al. 1982a, b). However, these were interpreted as signs of an extended metabolic capacity of lower motor neurons, rather than the presence of sympathetic co-innervation at NMJs. In one of these reports, muscle cross-sections were used, which showed clear, plaque-like TH immunoreactivity just next to staining of nicotinic acetylcholine receptors (nAChR) that were labeled by fluorescent α-bungarotoxin (Chan-Palay et al. 1982b). However, these samples did not allow to assign the TH immunoreactive signal to a corresponding axon. In other words, the exact nature of the TH signal remained uncertain. More than 30 years later, optical tissue clearing in combination with immunofluorescence staining and 3D confocal microscopy of diaphragm and hindlimb muscles of newborn, young and adult mice demonstrated that sympathetic neurons were indeed present in the immediate vicinity of NMJs (Khan et al. 2016; Straka et al. 2018, 2021). In diaphragms of newborn mice, TH-fluorescence signals ran alongside and within the synaptic band that is typically observed in this type of muscle (Straka et al. 2018, 2021). With increasing age, sympathetic innervation strongly ramified. Indeed, in adult diaphragm, sympathetic axons followed the larger blood vessels and then bifurcated at more or less regular intervals with one branch continuing to run along the blood vessel and another alongside the muscle fibers (Straka et al. 2021). This way, sympathetic, TH-positive axons traversed large parts of the muscle in width and height. Conversely, the axons of lower motor neurons showed a completely different pattern, i.e., these formed bundles directed exclusively to the synapse bands (Straka et al. 2021), where they branched locally to form motor units. A closer look at the distribution and morphology of TH-fluorescence signals demonstrated that sympathetic fibers apparently approach NMJs in a variety of rodent muscles, including facial muscles (Tereshenko et al. 2023), diaphragm (Khan et al. 2016; Straka et al. 2018, 2021), paw (Rodrigues et al. 2018) and hindleg muscles, like extensor digitorum longus (EDL) and tibialis anterior (Khan et al. 2016; Straka et al. 2018). Detailed analyses revealed the presence of plaque-like enrichments of TH-signals at the NMJ, which were sometimes complementary to the AChR postsynaptic labeling (Rudolf et al. 2013b; Khan et al. 2016; Straka et al. 2018). In quantitative terms, in mouse EDL muscle, TH-positive plaques at NMJs increased from roughly 40% of NMJs at birth to more than 80% in the adult (Straka et al. 2018). These data suggested that sympathetic co-innervation of NMJs is rather the rule than the exception, at least in some muscles, such as tibialis anterior and EDL. Three lines of evidence confirmed that the TH-staining in muscles was actually of sympathetic origin, i.e., retrograde tracing using fluorescent cholera toxin injected into either tibialis anterior or gastrocnemius (Rodrigues et al. 2018), anterograde tracing using DiI application at the sympathetic chain in transgenic reporter mice, and immunostaining of TH in noradrenergic neurons-reporter mice (Khan et al. 2016). In summary, these morphological data demonstrated a wide-spread distribution of sympathetic innervation in skeletal muscles in general, and of NMJs in particular.

## Experimental evidence for the functional relevance of sympathetic co-innervation at NMJs

**Fig. 1:**
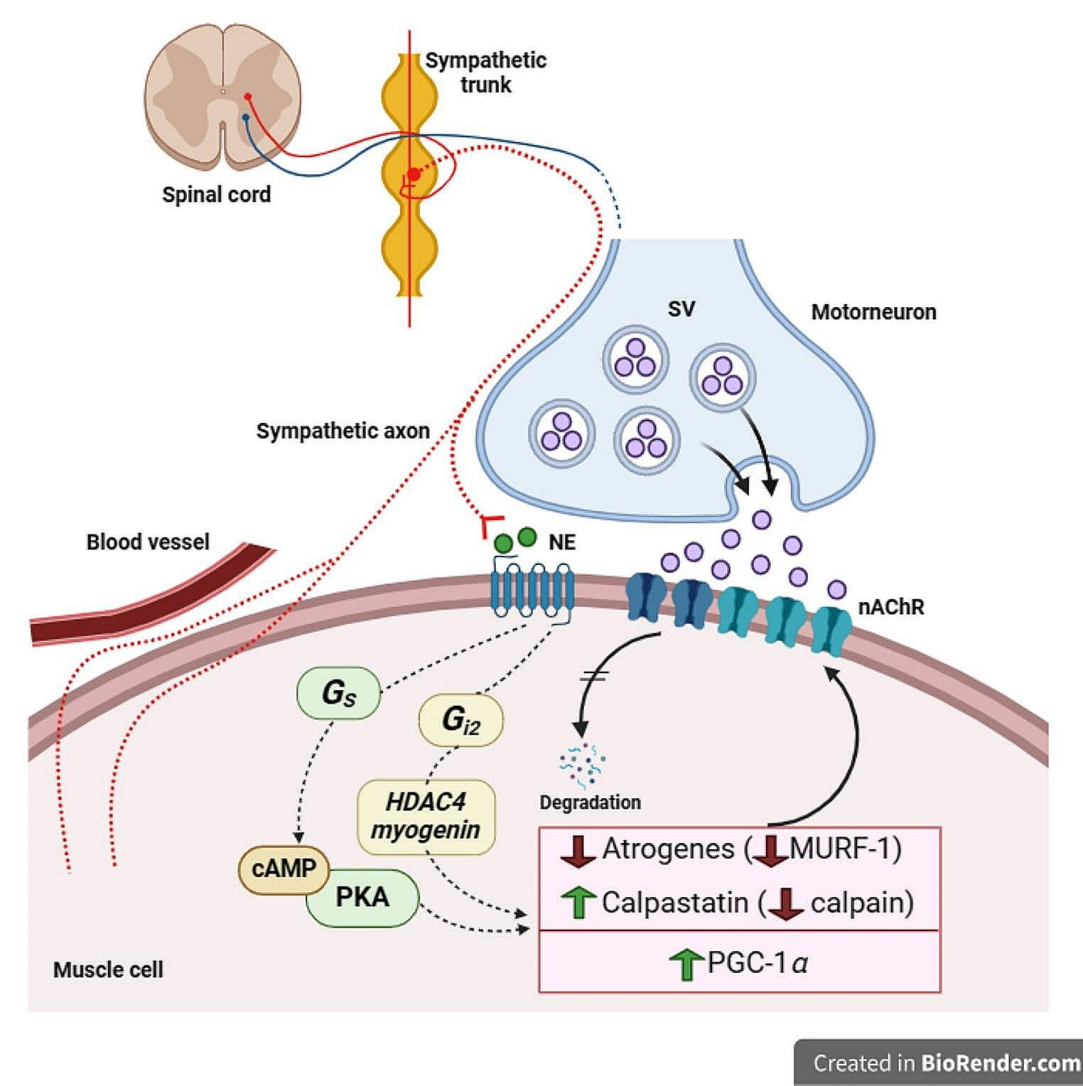
Sympathetic nervous system acts upon multiple targets within skeletal muscle tissue. The schematic drawing illustrates that sympathetic innervation in skeletal muscle as a functional unit affects not only vasomotor activity, but impacts also on muscle fiber and motor neuron physiology, e.g., by controlling muscle force production and synchrony of synaptic vesicle (SV) release, respectively. At the NMJ postsynapse, sympathetic release of norepinephrine (NE) affects the protein turnover in general and that of nicotinic acetylcholine receptors (nAChR), in particular. Mechanistically, this appears to involve downregulation of proteolytic systems (atrogenes, calpain). Control of these systems might occur via adrenoceptor-mediated tuning of cAMP/PKA and/or HDAC4/myogenin axes. In addition, sympathetic innervation positively affects PGC-1alpha signaling

To yield evidence for a functionally active sympathetic co-innervation at NMJs, gain-of-function and loss-of-function experiments were performed in different laboratories. Gain-of-function studies used either acute electrical or optogenetic stimulation of sympathetic ganglia, or pharmacological stimulation of adrenoceptors in combination with functional readouts. Indeed, two-photon microscopic imaging in tibialis anterior muscles expressing genetically-encoded fluorescent biosensors revealed a rapid rise in postsynaptic activity of β_2_-ARs, elevation of local cAMP levels, and cyto-nuclear translocation of the transcriptional co-activator, PGC-1α, shortly after electrical stimulation of paravertebral sympathetic ganglia (Khan et al. 2016) (Fig. 1). On the presynaptic side, classical studies have suggested that catecholamines act at the NMJ to synchronize vesicle exocytosis and augment the release of neurotransmitter (Bowman 1981). In agreement with this notion, recent studies have demonstrated that short-term electric or optogenetic stimulation of postganglionic sympathetic neurons led to an immediate increase of the frequency of miniature-endplate potentials, but did not alter amplitude or quantal content of endplate potentials (Wang et al. 2020a, b). In contrast, long-term injection of adrenoceptor agonists, salbutamol or clenbuterol, showed not only a significant enhancement of miniature endplate potential frequency but also of endplate potential quantal content in plantar nerve-lumbricalis preparations, being these effects dependent on extracellular Ca^2+^ (Rodrigues et al. 2019). Furthermore, pharmacological stimulation of adrenoceptors in mouse diaphragm muscles affected spontaneous and evoked release of acetylcholine in an adrenoceptor-dependent manner: while agonists of adrenoceptors α_1_ and β_1_ decreased spontaneous release and quantal content, β_2_-AR stimulation increased quantal content (Tsentsevitsky et al. 2020). Finally, norepinephrine and β_1_-agonists were found to synchronize evoked potentials in fatigued frog cutaneous pectoris and mouse diaphragm preparations (Bukharaeva et al. 2000; Tsentsevitsky et al. 2020). At difference, norepinephrine was not active in mouse soleus muscle, but epinephrine was (Khuzakhmetova and Bukharaeva 2021). Together, these gain-of-function data suggest that sympathetic tone affects pre- and postsynaptic signaling at the mouse NMJ in an acute and long-term manner, likely in a muscle-dependent and adrenoceptor-dependent manner. Regarding the precise mechanisms, the cell types involved, and the functional roles of different adrenoceptors, there is still some debate; please refer to recent reviews (Bukharaeva et al. 2021; Delbono et al. 2021). Altogether, gain-of-function data demonstrated acute pre- and postsynaptic roles of sympathetic co-innervation at the NMJ.

More long-term functions of sympathetic innervation at the NMJ were investigated by loss-of-function experiments that either involved local chemical or surgical sympathectomy. In the prior, intra-muscular injection of the neurotoxin, 6-hydroxy dopamine, over the course of two weeks led to a sustained, nearly complete deletion of sympathetic innervation of the treated muscle (Khan et al. 2016). Conversely, surgical sympathectomy at the level of the corresponding paravertebral L2/L3 sympathetic ganglia led to an almost instantaneous loss of sympathetic innervation of the entire hindleg (Silveira et al. 2014; Rodrigues et al. 2018). Both, chemical as well as surgical sympathectomy induced a similarly massive loss in muscle mass (Khan et al. 2016; Rodrigues et al. 2018), which was completely rescued by simultaneous systemic application of sympathomimetics, such as clenbuterol (Khan et al. 2016; Straka et al. 2021). Fittingly, also frequency and amplitude of miniature endplate potentials, amplitude and quantal content of endplate potentials, as well as induced muscle force were significantly reduced upon sympathectomy (Rodrigues et al. 2018). Notably, the loss in muscle force was more prominent upon stimulation of the motor nerve than upon direct stimulation of the muscles (Rodrigues et al. 2018), again suggesting an effect of sympathectomy on both, motor innervation and muscle fiber. While muscle fiber typing appeared to be unaffected upon sympathectomy, loss of cross-sectional area was observed in different fiber types, depending on which hindleg muscle was examined (Rodrigues et al. 2018). Furthermore, a careful analysis of motor neurons revealed a loss of neurofilament phosphorylation, an increase in relevant protein phosphatases types 1 and 2 A, a reduced axon diameter, and a larger variability of motor axon myelination (Rodrigues et al. 2018). Thus, loss-of-function data support a principal role of the sympathetic co-innervation for regulating NMJ-electrophysiology as well as motor neuron stability and muscle trophism.

## Role of sympathetic co-innervation in protein trafficking and turnover in muscle

At the level of NMJs, sympathectomy led to massive morphological alterations at both, pre- and postsynapse, which were mainly characterized by NMJ shrinkage and reduced ramification (Khan et al. 2016; Straka et al. 2021). These signs were accompanied by an increased number of postsynaptic endocytic vesicles containing nAChR and a reduced metabolic stability of these postsynaptic receptors (Straka et al. 2021). Transcriptomic and proteomic analyses of sham-operated vs. sympathectomized hindleg muscles supported strong effects of sympathectomy on the regulation of vesicle trafficking, in particular, an enhancement of endocytosis (Rodrigues et al. 2018; Straka et al. 2021). Fittingly, mechanistic studies identified a significant reduction of the ratio of surface/total nAChR upon sympathectomy, which again argued for a loss of synaptic nAChR under this condition (Rodrigues et al. 2018). Based on previous reports, which demonstrated a key role of the E3-ubiquitin ligase, MuRF1, in endolysosomal/autophagic degradation of nAChR (Rudolf et al. 2013a; Khan et al. 2014; Carnio et al. 2014; Wild et al. 2016), the regulation of surface/total nAChR upon sympathectomy was studied in MuRF1 KO-mice. Notably, here the effect of sympathectomy on the reduction of surface/total nAChR as found in wildtype animals was blunted (Rodrigues et al. 2018). Accordingly, this was accompanied by a transient rise in MuRF1 and HDAC4 expression at 3 days after surgical sympathectomy and of myogenin (Rodrigues et al. 2018), hinting to an earlier reported gene regulatory axis involving HDAC4/5 with myogenin, miR-206, and MuRF1 as downstream targets (Moresi et al. 2010). To make the link from SNS over G-protein coupled receptor signaling to this regulatory axis, the role of G_αi2_ was tested. Indeed, endogenous G_αi2_ was reduced upon sympathectomy and, fittingly, overexpression of constitutively active G_αi2_ in hindleg muscles blunted the rise of myogenin and the loss of surface/total nAChR upon sympathectomy (Rodrigues et al. 2018). In addition, also G_αs_-mediated cAMP/PKA signaling was found to be activated by the SNS and to participate in the maintenance of the NMJ (Fig. 1). Indeed, application of norepinephrine in vivo (Silveira et al. 2020) and in vitro (Silveira et al. 2014) activated PKA and reduced MuRF1 mRNA levels in rat skeletal muscles. On top of this inhibitory effect on E3-ubiquitin ligase activity, catecholamines regulated the abundance of nAChR through calpain, a calcium-dependent protease involved in neural insults and neurodegeneration (Groshong et al. 2007). In *Drosophila* development, calpain modulated synaptic function through the degradation of glutamate receptors GluRIIA, but not GluRIIB, at the NMJ (Metwally et al. 2019). In C2C12 myotubes, calpain induced dispersion of AChR clusters at NMJs, an effect that was inhibited by overexpression of its endogenous inhibitor, calpastatin (Chen et al. 2007). Accordingly, in a mouse model of the slow-channel myasthenic syndrome, where Ca^2+^ overload occurred at the NMJ due to mutations in the genes encoding AChR subunits, transgenic overexpression of calpastatin ameliorated the symptoms and neuromuscular transmission (Groshong et al. 2007). Under physiological conditions, the gene expression of calpastatin is increased by cold exposure in rat muscles, an effect that is abrogated by plasma depletion of epinephrine (Manfredi et al. 2017). Conversely, the activity and gene expression of calpastatin is increased after β_2_-agonists treatment (Bardsley et al. 1992; Goncalves et al. 2012), suggesting that this molecular mechanism underlies the inhibitory effect of SNS on calpain system, which could be implicated in the maintenance of NMJ. In consonance with this notion, cAMP-inducing agents including norepinephrine (Silveira et al. 2014), cAMP-phosphodiesterase inhibitors (Baviera et al. 2007) and β_2_-agonists (Goncalves et al. 2012; Gonçalves et al. 2019) suppress the autophagic/lysosomal and/or calcium-dependent proteolysis (calpains), two proteolytic systems that degrade nAChR (Khan et al. 2014; Wild et al. 2016). Similarly, also the pleiotropic neuropeptide, α-calcitonin gene related peptide (CGRP), is capable of inducing cAMP production in muscle (Machado et al. 2019), and to rescue the reduction of NMJ area in denervated muscles of rats via suppression of the endolysosomal/autophagic degradation of nAChR and calpain activity (Machado et al. 2019). In summary, these data argued for a homeostatic control of synaptic nAChR levels by the sympathetic innervation through G-protein coupled receptor mediated modulation of corresponding gene expression profiles (Fig. 1).

## Concluding remarks

Potential roles of sympathetic co-innervation at the NMJ in the etiology of neuromuscular disorders and in their therapy are manifold and have been discussed with respect to congenital myasthenic syndromes, myasthenia gravis, amyotrophic lateral sclerosis, spinal muscular atrophy, and sarcopenia in recent reviews (Cao et al. 2020; Rodríguez Cruz et al. 2020; Bukharaeva et al. 2021; Delbono et al. 2021; Mazzaro et al. 2023; Ohno et al. 2023). In many of these disorders, sympathomimetic drugs have been used for treatment, with variable success. Clearly, the facts that the SNS does not only innervate the NMJ but large parts of many skeletal muscles (Barker and Saito 1981; Khan et al. 2016; Straka et al. 2018, 2021; Rodrigues et al. 2018; Tereshenko et al. 2023), and that it uses (and is affected by) endocrine in parallel to neural signal transmission complicate the search for a precise understanding of causal and mechanistic relationships. Additional difficulties include the facts that (i) many adrenoceptors (and potentially further receptors, such as NPY receptors and purinergic receptors) with partially contrasting effects are involved (Bukharaeva et al. 2021) in the adrenergic effects, that (ii) the SNS is a major vasomotor control instance (Katayama and Saito 2019) that (iii) also affects skeletal muscle gene expression (Rodrigues et al. 2018; Straka et al. 2021) and (iv) acute sarcomeric force production (Sculptoreanu et al. 1993; Rudolf et al. 2006; Hotta et al. 2021). Finally, (v) sympathetic signaling molecules do not serve as bona fide neurotransmitters but as (neuro)modulators with volume transmission (Agnati et al. 1995), which renders the precise spatial attribution of norepinephrine release to its consequent cellular effects difficult. Regardless of these complications, research in the recent years has demonstrated important functions of the SNS in skeletal muscle that extend far beyond vasomotor control and future studies, for example, involving optogenetic approaches and adequate genetic mouse models, will further our insights into this fascinating field of systemic muscle research.
